# An approximation of flights, delays and costs for different forecast scenarios: A backcasting exercise

**DOI:** 10.1016/j.heliyon.2024.e26480

**Published:** 2024-02-19

**Authors:** I. Galarraga, L.M. Abadie, T. Standfuss, I. Ruiz-Gauna, N. Goicoechea

**Affiliations:** a Basque Centre for Climate Change (BC3), Sede Building 1, 1st Floor Scientific Campus of the University of the Basque Country, 48940, Leioa, Spain; b Metroeconomica, Spain; c UPV/EHU, Spain; d Technische Universität Dresden, Institute of Logistics and Aviation, Germany; e Escuela de Ingeniería de Bilbao, Universidad del País Vasco-Euskal Herriko Unibertsitatea (UPV-EHU), Ingeniero Torres Quevedo Plaza 1, 48013, Bilbao, Bizkaia, Spain

**Keywords:** Cost of delays, Backcasting exercise

## Abstract

Air Navigation Service Providers (ANSPs) play a critical role as a natural monopoly within the air traffic system and are subject to regulation. Achieving preset performance targets necessitates efficient resource planning, contingent upon accurate traffic forecasts. This means that forecast precision is a key determinant of operational efficiency. In this study, we employ backcasting techniques to gauge the influence of forecast errors on air traffic management performance. This is done for eleven airspaces and seven years of data. The paper seeks to estimate the cost of delays if the actual number of flights for the period 2015–2020 had been as predicted by EUROCONTROL through its specialised service STATFOR. To assess the impact of forecasting errors, we analyse the discrepancy between the predicted and actual figures for flight data, specifically focusing on delays. Our results show that forecast errors have a noteworthy, adverse effect on performance. Inaccurate predictions prevent efficient resource allocation. We prove that a marginal increase in forecast quality would significantly reduce overall costs for stakeholders.

## 1 Motivation

### 1.1 ANSP performance

In 2019, air traffic across the European Civil Aviation Conference (ECAC) network increased to an all-time high of more than 11 Mio. flights. In that same year, huge efforts were made to improve overall network performance, as 2018 had proved to be the worst year of the previous ten for Air Traffic Flow Management (ATFM) delays and flight cancellations, with almost 25.5 million minutes attributed. Flight delays entail costs of millions of Euros for airlines, passengers and Air Navigation Service Providers (ANSPs), so better management of these inefficiencies is needed. Despite the strong impact of the COVID-19 pandemic on air transport, demand is expected to increase in the medium to long term, leading to traffic levels similar to those of 2019.

The main goal of benchmarking is to enhance efficiency. In the light of this industry's market dynamics, benchmarking efforts frequently concentrate on assessing the performance of air navigation services. These services play a critical role in ensuring the safety and seamless management of flight operations. They operate as natural monopolies and fall under political regulation. EUROCONTROL has established four Key Performance Indicators (KPIs), including “cost-efficiency” and “capacity,” along with specific targets such as minimising delays, to monitor performance.

Traffic forecasts play a crucial role in the cost and resource planning of an Air Navigation Service Provider (ANSP). Underestimating traffic can lead to delays, especially in airspace that is already saturated, but overestimating it results in higher unit costs (i.e. € per flight hour) and reduced cost efficiency. In consequence, ANSPs are faced with a trade-off between cost efficiency and capacity.

A previous study [1] has demonstrated that a significant number of ANSPs struggle with mispredictions, which can result in inefficiencies, particularly in terms of underestimating flight volumes, which in turn leads to delays. However, it is important to note that this partial inefficiency cannot be attributed to air traffic control, as it is entirely exogenous to its operations. Therefore, the primary recipients of any potential improvement efforts would be the forecasters.

### 1.2 STATFOR, responsibility, scenarios

EUROCONTROL,^1^ the pan-European, civil-military organisation that supports European aviation, produces regular statistics through its specialised service STATFOR.^2^ We specifically focus on the 7-year forecasts published every spring and autumn, which involve three scenarios: Baseline, High and Low. The range between the high and low scenarios can be interpreted as a confidence interval, representing the range of possible outcomes.

It is important to note that ANSPs need to plan capacity and costs based on one of these scenarios. If the scenarios are not borne out in reality, cost increases usually result. In particular, in the case of underestimation the probability of delay is very likely to increase, depending on other airspace and traffic characteristics (such as saturation and complexity), and with it the associated costs.

[1,2] illustrate that there may be significant deviations between forecast and actual figures, and argue in favour of efforts to improve forecasting quality, especially regarding demand for flights. Despite a wide confidence interval, most forecasts are not borne out in the actual number of flights, i.e. actual flight numbers are higher than the high scenario or lower than the low scenario. These results are confirmed by applying metrics for forecast quality (such as the Mean Absolute Percentage Error (MAPE) score). We show that such deviations may have significant implications for the stakeholders of the air traffic system.

The STATFOR 2014 report forecasted traffic growth at European level of 2.4% for 2015 (baseline scenario^3^), 2.8% for 2016, 2.3% for 2017, 2.3% for 2018, 2.8% for 2019 and 3.1% for 2020. However, unexpected, unplanned-for demand for flights resulted in substantial delays and thus significant financial costs for ANSPs. Europe’s air traffic grew by 4.3% from 2016 to 2017 and by 3.8% from 2017 to 2018. This means that STATFOR’s estimates of air traffic were rather low and air traffic authorities required ANSPs to plan accordingly.

### 1.3 Literature review: backcasting method

This study seeks to estimate what the cost of delays would have been if the actual number of flights for 2015 to 2020 had been as forecast by STATFOR, i.e. to analyse what delays and subsequent costs may be caused by low-quality forecasting or, to put it another way, by a negative deviation between forecast and actual traffic. This provides a better understanding of what costs can be attributed to unexpected increases in air traffic. This approach is commonly known as backcasting. It is framed within scenario planning literature [[3], [4], [5], [6], [7]]. Forecasting predicts the (unknown) future based on current trends while backcasting analyses the future from the opposite direction. It starts by envisioning desired future conditions and then works backward to identify specific policies and programmes that connect that specified future to the present, rather than extrapolating present methods into the future [[8], [9], [10], [11], [12], [13], [14]].

This method has been used in the field of transportation for sustainable transport and transport planning [[15], [16], [17], [18]]. It enables alternative scenarios to be studied based on a sound methodological approach. However, to our knowledge, to date there have been no such studies on air transport,^4^ so this paper fills that gap and represents a considerable advance in that direction. The scope of the paper is limited to saturated ANSPs underestimating traffic forecasts in Reference Period 2 (RP2), which covers 2015–2019.^5^

The paper is organised as follows: Section 2 presents the data used in the study. Section 3 details the methodology and Section 4 sets out the main results. Section 5 deals with the limitations of the method while section 6 concludes and suggests further research.

Please note that we use “number of flights” and “demand” as synonyms. There may be differences between them, but we use this simplification for the sake of illustration.

## 2 Data and transformation

### 2.1 Operational data

To assess the impact of forecasting errors, we analyse the discrepancy between the predicted and actual figures for flight data, focusing on delays. This involves comparing the delay that would have occurred if the prediction had been accurate with the actual observed delay. To carry out this analysis effectively, we require three essential data points:

• Predicted flights, i.e. the number of flights forecast for a given period, as published by STATFOR.
• Actual flights, i.e. the real, observed flights that took place during the same period. These data are obtained from PRU.
• Actual delay, i.e. the actual amount of delay experienced by flights during the specified period, as recorded and measured in terms of time.

We use the STATFOR spring reports^6^ as our source for forecast flights. However, these forecasts are country-specific. The performance targets regarding capacity and cost efficiency are ANSP-related, so the forecast data need to be transformed from ‘flights per country’ into ‘flights per ANSP’. We do this by using data from the Performance Review Unit (PRU), which are available on EUROCONTROL's One Sky portal. These data are used for official performance reports, such as [23].

**Fig. 1:**
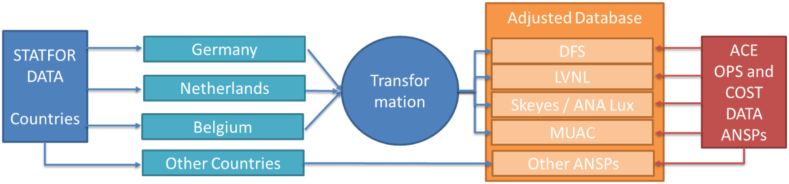
Data transformation into ANSP-related flights (Source: own work).

As shown in Fig. 1, the growth rates (forecast by STATFOR) are applied to the ANSP-related flights (provided by PRU) to calculate the forecast flights per ANSP [24]. The resulting figure is compared with the actual numbers of flights (again based on PRU data). For Maastricht Upper Airspace Control (MUAC) we use the average of the growth rates forecast for the countries covered (Germany, the Netherlands and Belgium).

The analysis of delays is based on PRU data with no post-op adjustments [25]. The database contains data on flights, delays (in minutes), delay causes and the number of delayed flights. Delay is split into several causes, which makes it possible to filter out delays that are not attributable to ANSPs (e.g. weather). In accordance with the identification letters used by EUROCONTROL, causes are described as “CRSTMP”^7^ delays. Capacity targets stipulate that the total ATFM delay per flight must not be greater than 0.5 min in pan-European air traffic. However, ANSP-specific targets are lower because most flights use multiple airspaces (see also Section 3). These targets are published by some ANSPs for both the overall delay and the CRSTMP delay.

**Fig. 2:**
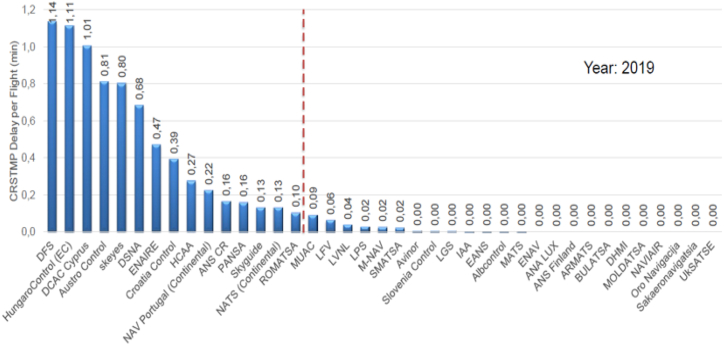
Saturated and non-saturated airspaces in Europe (Source: own work).

Once this information was collected, saturated airspaces in Europe were identified by following two criteria: 1) average delay minutes (ADM) of 0.1 min or more per flight; and 2) checking whether a high figure for average delay minutes was caused by extreme data. This analysis was conducted using boxplots to show the scattering of daily delay, including median, mean, quartiles and outliers. A large difference between mean and median and long whiskers (or outlier points) indicate a high degree of scattering and suggest that the ADM figure might be caused by an extreme figure (and not by saturated airspace). Fig. 2 shows the average CRSTMP delay for all ANSPs considered. Those to the left of the dashed line are considered to be saturated.

The methodology for selecting saturated airspaces is subject to debate. It is important to acknowledge that many ANSPs cover a large number of sectors, so the representation of flights and delays may only provide average values. Airspace oversaturation and the accumulation of delays may be concentrated in space and time. However, we consider this approach to be valid for several reasons: firstly, benchmarking is primarily conducted at ANSP level and there are no specific target values at sector level. Secondly, the selection of airspaces for backcasting is based on the inclusion of relevant data. Lastly, data availability is often limited to ANSP level, making it practical to analyse and assess performance at that level.

### 2.2 Costs of delay

The first attempt to calculate the monetary cost of one minute of delay, as incurred by airliners in Europe, was made by the Transport Studies Group at the University of Westminster [26]. Delays were measured relative to the last flight plan filed, with average costs of 72 Euros per minute of delay. Successive revisions [27,28] were published which included more aircraft types, a gate-to-gate perspective on delay costs and updates on the average cost of delays. The latest revised cost of one minute of delay is 104 Euros, set by the Performance Review Unit in 2019 [29].

However, average values do not seem to be a good representation of real costs, as not all flights are subject to the same delay (i.e. for the same total amount of delays in a given period of time there may be many flights with very short delays or just a few with very long delays), so it is crucial to determine the distribution of delays in order to fully comprehend how they affect the total cost.

By extending the analysis to ten delay classes with a different cost attributed to each class, we can significantly improve the cost calculations. We also estimate a mathematical function for all these cost ranges, which we then apply to the distribution of delays in an attempt to enhance the accuracy of cost calculations^8^ or costs from a climate perspective due to air management [31].

## 3 Methodology

### 3.1 General approach

Analysing forecasting errors entails applying two complementary approaches, which together provide a comprehensive understanding of the situation. These approaches are executed in three fundamental phases to ensure a thorough examination of the data.

1. Flight Analysis: in the first phase, the analysis focuses on studying actual and planned flight data. This involves comparing the number of flights that were forecast or planned with the actual number of flights during the given period. Examining these data reveals any disparities or deviations between the forecast and observed flight volumes.
2. Backcasting Delays: the second phase involves a backcasting exercise specifically applied to delays. Backcasting entails determining how much delay there would have been if the forecasts had been accurate. By comparing this backcasted delay with the actual observed delay, the impact of forecasting errors on delays can be determined. This analysis provides insights into the scale and consequences of these errors as determined in phase 1.
3. Cost Analysis: the third phase focuses on assessing the costs associated with the backcasted delays. By quantifying the financial implications of these delays, a comprehensive understanding of the economic impact of forecasting errors can be obtained.

In the first approach, daily flights are clustered into value ranges. By examining the observations (i.e the number of flights of a specific ANSP on a specific day in the specific cluster), it is possible to estimate the probability of exceeding the performance target. The primary objective is to assess the feasibility of the capacity targets specified and determine what clusters (daily flight movements) may be considered unrealistic. Backcasting and delay cost analyses serve as complementary investigations to further enhance the understanding of the situation.

### 3.2 Approach 1. clustered delay analysis

Performance targets are an essential part of economic regulation^9^ in Europe. For capacity, the EU-wide delay target is 0.5 min per flight. However, individual targets per ANSP or Functional Airspace Block (FAB) are lower since most flights are controlled by multiple ANSPs or FABs. These targets are based on performance plans. Thus, in our first step we set out to find the probability of these performance targets being missed depending on the flights.

We expect the probability of performance targets being missed to increase as traffic increases. To calculate these probabilities, we use a methodology that classifies flights into specific value ranges. By using value ranges, we can analyse the link between traffic volume and the likelihood of missing performance targets^10^. To ascertain the likelihood, we examine all days where the demand aligns with the specific cluster and observe how frequently the delay target was exceeded. That number is then checked against the total number of observations within that cluster. To ensure robustness, we shift boundaries and classes to perform sensitivity analyses. The analysis is run for each ANSP separately.

**Fig. 3:**
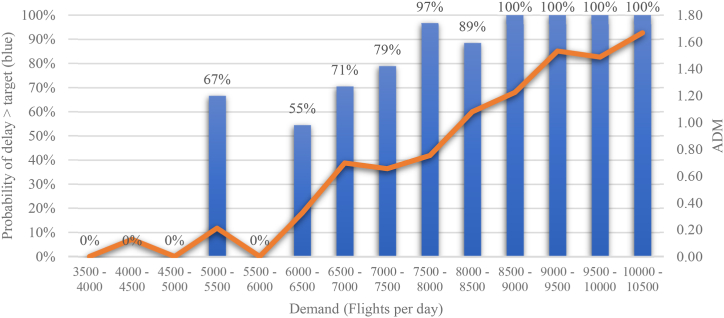
Average Delay Minutes and Probability of Delay Target Mismatch, DFS 2019 (Source: own work).

Fig. 3 illustrates the analysis conducted on German DFS, where we calculate both the average delay minutes (ADM) and the probability of the delay per flight exceeding a specific limit for each group (cluster). The example given indicates that if the daily demand exceeds 8500 flights there is a 100% probability of the delay target being exceeded. Even when the daily demand is between 6000 and 6500 flights, more than 50% of flights experience delays that exceed the specified target. Additionally, as daily demand increases the average delay minutes also increase, which is not surprising. It is important to note that this investigation focuses specifically on en-route delay targets. Please note that the extreme value for the “5000–5500 flights” cluster is due to a low number of observations (1). Subsequently, it might be assumed that this day was characterised by a significant external shock.

For the backcasting, we need to ‘adjust’ demand to the number of flights forecast by STATFOR. This adjustment is made for each day and is based on certain restrictive assumptions:

• The reduction in flights is distributed evenly over the year.^11^
• There is no spatial shift of traffic flows.
• The average delay in minutes is constant for each class (it does not change because it might be seen as a proxy for capacity).
• Note that STATFOR also uses historic city pairs for forecasts and increases them to get overflights. This gives rise to the same restriction as in the forecast.

**Fig. 4:**
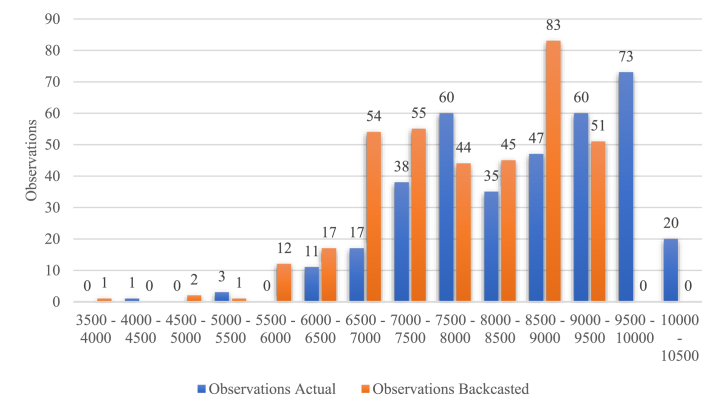
Shift in Observations, DFS 2019 (Source: own work).

**Fig. 5:**
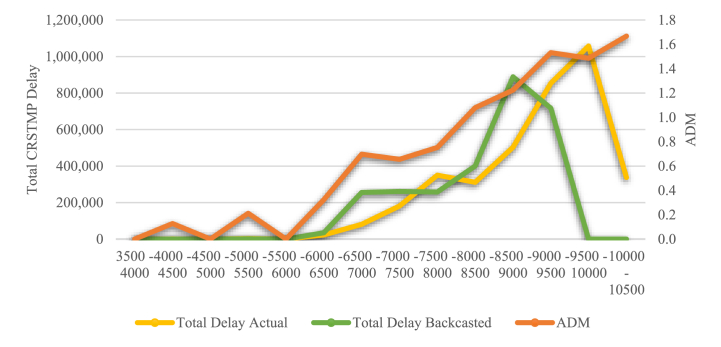
Actual and Backcasted Delays, DFS 2019 (Source: own work).

Adjusting the flights might result in redistributing observations across different clusters. Essentially, this redistribution dilutes the distribution of observations, shifting the number of flights towards alternative value ranges. This trend is illustrated in Fig. 4 for German DFS, where the blue columns depict the actual values and the orange columns the backcasted ones. Given the constant capacity, denoted by the specific ADM of the cluster, we determine the (backcasted) delay by multiplying the number of observations by the average delay minutes. Consequently, if observations (flights per day) move to a lower cluster through backcasting, the overall delay decreases. This occurs because the observations are then multiplied by the (lower) ADM value. Consequently, the corresponding delay curve shifts to the left (see Fig. 5). Once the (backcasted) total delays have been calculated, the total costs and the costs per flight can be determined.

The ADM curve further helps to estimate the functional form of the link between delay and demand. One possibility is a linear increase after an offset, on the basis that there is no delay if flights do not exceed a certain threshold. However, this assumption is debatable, as delays can occur even when traffic is low. We therefore assume that there is an exponential link.

**Fig. 6:**
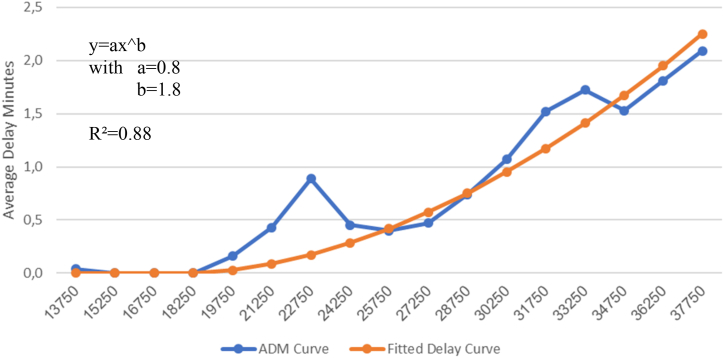
Exponential Flattening of the curve, Europe 2019 (Source: own work).

Fig. 6 shows the ADM curve (blue) for aggregated data (European airspace). As discussed above in the context of Fig. 3, observations per group may differ and may have extreme values due to a low number of observations. Subsequently, the ADM curve may have some peaks which are probably due to external shocks but not to operational limitations. These peaks may lead to a bias in calculating the delay and subsequently the corresponding costs.

To prevent this, we apply a mathematical flattening, using the functional form (1):

(1) $$y=ax^{b}$$

where y = delay, x = number of daily flights, and a and b are parameters for optimisation. In other words, we find the exponential function that best fits the observations (minimal quadratic deviation between observations of blue and orange dots).^12^ In addition to the functional form, it may be assumed that the flattened curve should not indicate a delay if the actual delay is zero. The orange curve in Fig. 6 shows the result for Europe after the exponential fitting.^13^ Note that the values on the x-axis represent the mean between the cluster boundaries.

What we are doing here is to use an exponential function to better fit the data distribution and compare results with both (a) clustered data and (b) using non clustered data together with an exponential function to explain the functional form.

If this procedure is applied to ANSP data, the deviating delay costs can be determined more precisely. As shown in Table 1, the backcasted delay is much lower than the actual delay. However, peaks in the ADM curve lead to some distortions. After the curve is flattened, the delay is slightly greater than the backcasted figure. Assuming €104 per minute of delay [[26], [27], [28], [29]] gives the overall delay costs. Table 1 shows the average CRSTMP delay minutes per flight and the subsequent average costs per flight.

**Table 1:** Delay and Costs for DFS, 2019 (Source: own work).

	Actual	Backcasted
Delay	3,702,239	2,740,061
Costs	€392,437,334	€298,504,081
Average CRSTMP delay/flight	1.19	0.96
**Delay costs/flight**	**€125.85**	**€104.09**

### 3.3 Approach 2. exponential flattening on non-clustered data

For this second analysis, a different method is proposed: (a) daily flights and delay minutes are used; (b) clustered delay analysis is omitted; and (c) exponential flattening is applied to the non-clustered data. The first approach is highly valid for providing a general approximation, but these three points significantly enhance the accuracy of the estimates.

More specifically, data on backcasted flights from the previous model are used to later relate the actual number of flights to the minutes of actual delay via function (1). Note however that the coefficients estimated are different from those in the previous approach.

Backcasted daily delays (in minutes) (BD) for all ANSPs and years are then estimated and divided by the number of delayed flights to give the backcasted mean delay (in minutes). Backcasted delays are thus explored via a different function for each year following an exponential flattening. An example of coefficients a and b, and of the model as a whole, is shown in Table 2 (results for the other ANSPs are shown in Appendix A).

**Table 2:** Coefficients and goodness of fit, DFS, 2015–2019 (Source: own work).

DFS	2015	2016	2017	2018	2019
**a**	2.12E-09	3.99E-28	7.6E-24	2.21E-16	4.87E-11
**b**	2.981	7.816	6.792	4.988	3.634
**Goodness- of-fit**	SSE: 8.605e+08 R-square: 0.04397 Adj. R-square: 0.04133 RMSE: 1540	SSE: 1.243e+09 R-square: 0.3672 Adj. R-square: 0.3655 RMSE: 1848	SSE: 4.004e+09 R-square: 0.443 Adj. R-square: 0.4414 RMSE: 3321	SSE: 8.375e+09 R-square: 0.6149 Adj. R-square: 0.6139 RMSE: 4803	SSE: 8.632e+09 R-square: 0.4491 Adj. R-square: 0.4476 RMSE: 4876

SSE (Sum Squared Error) and RMSE (Root Mean Square Error) are measures of goodness-of-fit. ${\;R}^{2}=1-\frac{SSE}{SST}$, where SST is the sum total of squares, i.e. the squared differences between the observed dependent variable and its mean. This process is applied to the rest of the ANSPs.

Note that in many cases the R-square may be low, for two main reasons: first, there is high volatility in daily data and no clusters. When cluster analysis is used data volatility is ignored by calculating average values. This means that above-average values may be offset by below-average values. However, in clustering a great deal of information is lost and the analysis may be more limited (note that in some ANSPs there are 0 min of delay on many days). In the link between delays and flights one part of the behaviour (i.e. the deterministic part) can be easily predicted and the other (i.e. the stochastic part) cannot. With the existing data, only the deterministic part can be calculated, which means that certain non-predictable information (such as strikes, etc.) is not considered in the analysis.

By contrast, when the actual and backcasted daily delay data are grouped into clusters, the R-square is much higher. However, it can be shown that working with daily data is much more suitable and enhances the analysis. For example, if the daily data in 2018 for DFS are clustered the R-square is 0.9804, which is greater than that obtained in Approach 1, where no daily data were used. This confirms that it is more appropriate to work with daily data, as it gives a better forecast than when clusters are used directly.

**Fig. 7:**
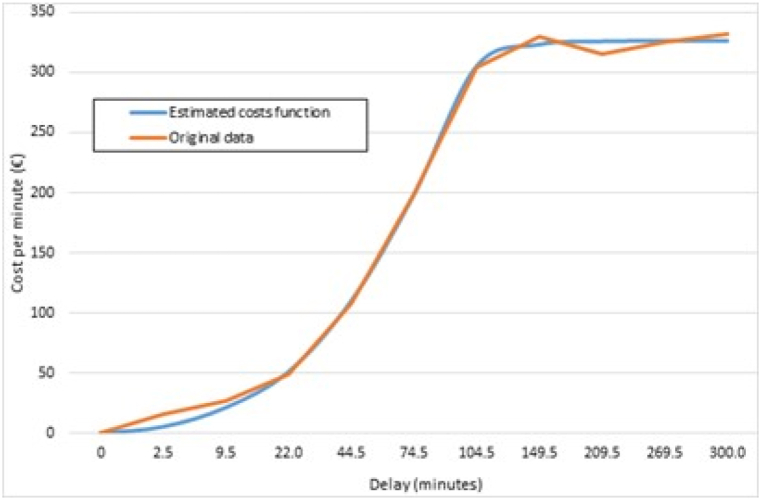
Cost functions and original data (Source: own work).

We now analyse the costs of delays. In this case we use the information on costs per minute from the study by the University of Westminster [28] rather than the average value of €104. These cost data are given for 10 delay ranges, which basically means that the cost varies significantly with the number of minutes accumulated. For example, when the delay is between 1 and 4 min the cost is €16.12 per minute. That figure rises to €336.94 per minute when the delay is greater than 300 min (See Fig. 7). The use of these cost ranges enables us to significantly increase the quality of our cost estimates. This is done in 2 steps using the data in Table 3 below (see Appendix B). Step 1 consists of estimating the cost functions (2) and (3) that mathematically fit the data in Ref. [28]:

(2) $$\text{For}\;\text{delays}\leq 74.5\;\text{minutes}\;\alpha_{1}y+\beta_{1}y^{2}$$

(3) $$\text{For}\;\text{delays}\;>\;74.5\;\text{minutes}\;\alpha_{2}y^{\beta_{2}}+\gamma$$

where y stand for delays and the annual parameters are $\alpha_{1}$, $\beta_{1}$, $\alpha_{2}$, $\beta_{2}$ and γ.

**Table 3:** ATFM delay ranges and weighted costs (per minute) in 2015 Euros (Source: own work based on [27,28]).

Delay range (min)	01–04	05–14	15–29	30–59	60–89	90–119	120–179	180–239	240–299	300+
Average total cost (Euros)	39.50	259.25	1074.02	4.78	14.74	31.55	49.02	65.70	86.97	99.09
Average cost per minute (Euros)	15.80	27.29	48.82	107.36	197.85	301.95	327.91	313.60	322.71	330.32

Step two is to apply these cost functions to the number of minutes of actual and backcasted delays. Table 3 below shows that the cost functions estimated fit the real data closely.

The original cost data refer to the figures for the different ranges from the University of Westminster study, i.e. minutes of delay multiplied by cost per minute, where estimated cost functions refer to the results from applying functions (2) and (3).

## 4 Results

Results are shown for ten saturated airspaces (DFS, DSNA, ANS CR, Austro Control, Croatia Control, DCAC, NATS, NAV Portugal, Skeyes and Skyguide) plus the Maastricht Upper Area Control Centre (MUAC), which manages the upper airspace (from 24,500 to 66,000 feet) over Belgium, the Netherlands, Luxembourg and the north-west of Germany.

### 4.1 Flights

As shown in Table 4, actual flights (i.e. the number of flights that actually take place each year) were higher than expected. The largest difference recorded is for NAV Portugal and the smallest is for ANSCR. The differences are in the range of 4–8% in most cases.

**Table 4:**
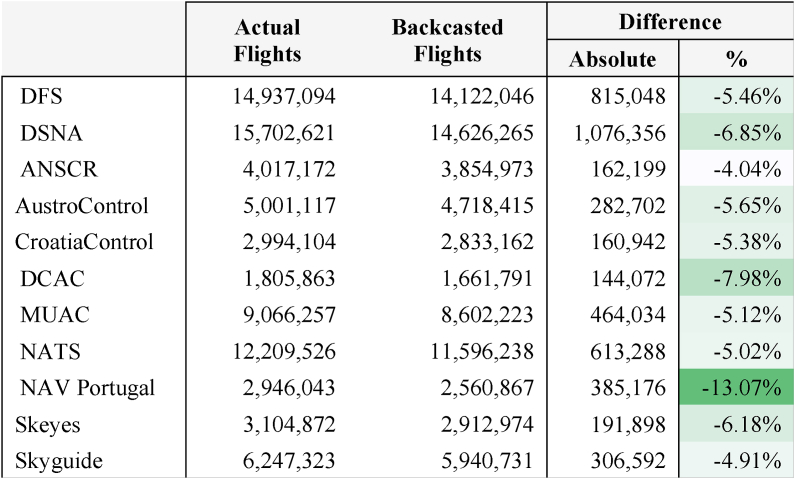
Actual and Backcasted Flights and difference in absolute and percentage terms for period 2015–2019 (Source: own work).

Differences are increasing over time in most ANSPs and range from 0.17% for Austro Control in 2016 to 17.66% for Nav Portugal in 2019 (see Appendix C).

### 4.2 Delays

Backcasted CRSTMP Delay Minutes are lower than actual CRSTMP Delay Minutes. This makes sense as actual flights exceeded the backcasted figures and, in general, the more flights there are, the more minutes of delay there will be. However, note that the exponential shape of the curve means that the difference in percentage terms between the two delays is greater than for the number of flights (see Table 5), whichever approach is applied (i.e. clustered analysis or non-clustered daily data). That is, the delays increase by proportionally more than the number of flights. In particular, the deviations between actual and backcasted delays exceed 21% for all ANSPs.

**Table 5:**
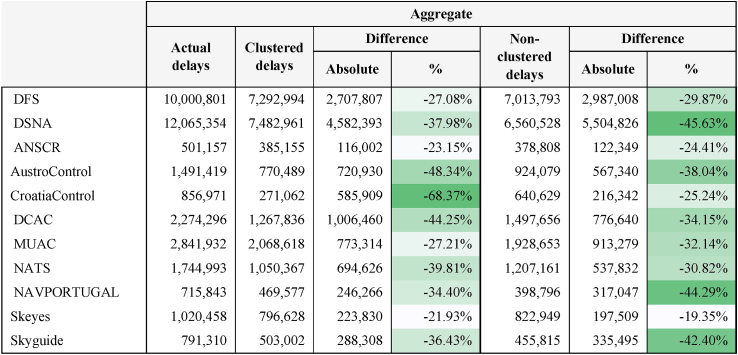
Difference between Actual and Backcasted CRSTMP Delay Minutes, in aggregate terms, applying clustered analysis and non-clustered daily data.

The results for each year are shown in Appendix D.

### 4.3 Cost of delays

Because backcasted delays are lower than actual CRSTMP delays, the cost of delays is also lower. The reasoning is that actual delays are caused mainly by the under-provision of services due to forecasts underestimating flights. This is referred to as “costs due to underestimation”. When delays are higher, the cost may also increase due to the distribution of delays.

Table 6 shows aggregate actual and backcasted delay costs for both the clustered analysis (i.e. using a figure of €104 per minute of delay) and the non-clustered daily data (using the mathematical functions). The results for each year are shown in Appendix E.

**Table 6:**
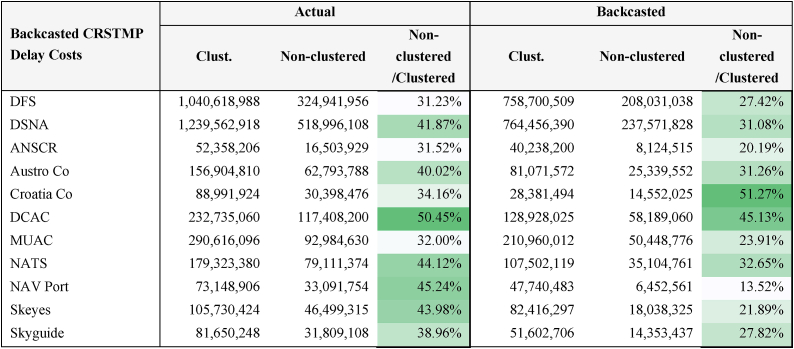
Actual and backcasted delay costs for the clustered analysis and the non-clustered daily data.

The Non-clustered/Clustered column depicts how representative the cost obtained with the non-clustered daily data is with respect to the clustered analysis. For example, when the functions are used the actual delay cost for DFS is 31.23% of the cost obtained using the average of €104 per minute. This is tantamount to saying that the average of the Actual Cost column overestimates the cost by 68.77%. The same applies to backcasted delay costs, albeit to a lesser extent. This makes perfect sense as there are many delays of a few minutes that are automatically overestimated when the average cost is used instead of the much lower cost given by Refs. [27,28]. This is why we argue that the accuracy of estimates increases very significantly when the cost functions and the full distribution of the delays are used, with lower than average costs being assigned for delays of a few minutes and far higher than average costs when delays are longer.

In short, it can be seen that there are greater differences between the clustered analysis and the non-clustered daily data because the former uses the average figure of €104, regardless of whether delays last, for example, 1 min or 70 min. By contrast, the exponential flattening of non-clustered data applies the cost functions. Results show that when mathematical functions are used the cost per minute is approximately 30.37% of the average figure of €104 (see Table 7). Considering that a delay of around 45 min is needed for the average cost per minute of delay to be €104, using the average can be seen as overestimating the cost of delays, given that there are many delayed flights with delays of less than 45 min (i.e. there are many delays of a few minutes that incur very low costs).

**Table 7:** Overall results (Source: own work).

Total 11 ANSPs and 5 years	Actual		Backcasted		Differences		%	
	Clustered	Non-clustered	Clustered	Non-clustered	Clustered	Non-clustered	Clustered	Non-clustered
**Total Cost (€)**	3,541,640,960	1,354,538,637	2,301,997,807	676,205,878	1,239,643,153	678,332,759	153.85%	200.31%
**Cost/minute (€)**	104	39.49	104	30.98	0	8.51	0%	127.47%

### 4.4 Overall results

When the 11 ANSPs and 5-year results are aggregated, it emerges that the low quality of the forecasts results in an additional cost of €1.24 billion (an increase of 154%) under the clustered analysis. When the non-clustered daily data are used, the additional cost is only just over half that figure at €678 million (an increase of 200%). The average cost per minute is also greater, with an increase of 127% (see Table 7).

**Fig. 8:**
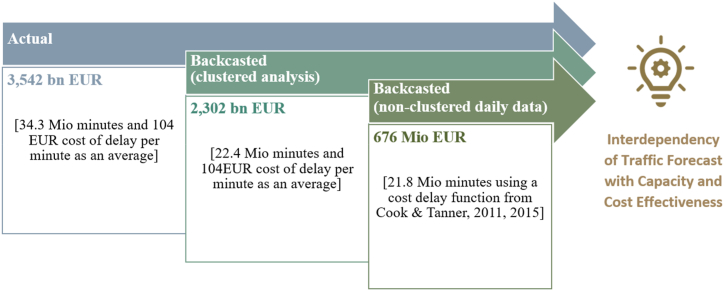
Overall results.

There are two effects which lead to the different results in the cost of delay of 3.5bn EUR and 676 Mio EUR. First, part of the delay is attributable to traffic forecasts. Some 12.7 Mio minutes are therefore not attributable to ANSPs although they still occur for airspace users. Second, the average cost of delay per minute decreases from €104 to €31 due to shorter delays and greater precision in calculating values. (See Fig. 8).

## 5 Limitations of the study

This study has several limitations related to some of the assumptions that had to be made for the analysis describe in pages 9 and 10. These could be discussed and would affect some of the results of this paper, but in any case, the method proposed is still a valid, robust technique for such analyses.

The problems in accessing sectoral data are another caveat worth mentioning in this paper. Opening up access to data sources such as EUROCONTROL could significantly enhance the accuracy of the estimates.

## 6 Conclusions

By using backcasting it is possible to estimate the number of additional delays caused as a consequence of low-quality forecasting by STATFOR or, in other words, by negative deviations between forecast and actual traffic. This is mainly because of the underestimation of services that results from the forecast number of flights being lower than the number of flights that finally occur.

In this study we first identify saturated airspaces in Europe and then determine the years in which there has been underestimation. Forecasts are transformed into ANSP-related flights, flights are then clustered into classes and average delay minutes and the probability of delay target mismatch is calculated. This approach gives a rough calculation of the CRSTMP delay induced by forecast bias (the number of flights forecast is lower than the actual number of flights). However, the actual contribution to CRSTMP delay might differ from the figures shown, as the reduction in flights shown is evenly distributed over the days. This means that there is no consideration of seasonal effects and no potential changes in traffic flows are considered. Thus, there is no consideration of spatial effects.

Using the ANSP level for performance assessment entails certain limitations. Delays can occur in specific sectors of the airspace and may vary over time, suggesting that a more granular approach is needed. However, we argue that performance assessment and target setting generally focus on ANSP level. ANSPs also have decision-making power, particularly regarding resource allocation. Data availability is another factor to consider. Currently, publicly available data are aggregated at ANSP or state level, making them easier to access and analyse at those levels. Accessing sector-level data would require collaboration with organisations such as EUROCONTROL, which possess the necessary data resources.

We therefore also propose an alternative method for estimating these figures comprising three steps: a) econometric estimation of functions (for each ANSP and year) linking the number of flights and delays; b) cost calculation, for which we estimate an econometric (mathematic) function for the costs of delay based on the study by Refs. [27,28]; and c) application of the cost function to estimated delays considering the full distribution of delays, i.e. for every ANSP and year. This makes it possible to significantly increase the accuracy of the estimates and provides lower estimates of the overall costs of delays.

This last method makes two improvements on earlier methods. Eliminating the cluster analysis means that functions linking flights to delays can be better estimated, as daily data are used. Applying the cost function also means that the accuracy of the cost estimates improves very significantly, as the cost range information estimated by Refs. [27,28] is used.

Results show significant increases in delays due to low-quality forecasting, resulting in major financial costs. Likewise, the cost of delay estimated using the non-clustered daily data is significantly lower than that estimated via the clustered analysis. As argued, this is mainly due to increased accuracy of estimation.

From a total financial cost viewpoint, forecast inaccuracies and their consequences need to be taken into account as the attributable costs of delays are significant and may be higher than costs for spare capacity. Unused capacity may therefore still be cost-optimal as it may pay off for airspace users, passengers and the functioning of the network. This is not considered here in terms of reactionary delay.

In the future, it would be useful to explore the possibility of analysing sectoral data using tools such as NEST. This would involve obtaining sector-level data from relevant authorities or organisations. However, it should be noted that accessing and utilising sector-level data would require data to be provided by EUROCONTROL or other relevant sources.

## Authorship contribution statement

All the authors listed have significantly contributed to the development and the writing of this paper.

## Data availability

All data can be downloaded from www.eurocontrol.int/dashboard/statfor-interactive-dashboard. And can also be made available upon request to the authors.

## Funding

This research is supported by María de Maeztu Excellence Unit 2023–2027 Ref. CEX2021-001201-M, funded by 10.13039/501100004837MCIN/10.13039/501100011033AEI/10.13039/501100011033. Further support is provided by the Spanish 10.13039/100014440Ministry of Science, Innovation and Universities (10.13039/501100003329MINECO) (Grant RTI 2018-093352-B-I00). Ibon Galarraga and Nestor Goicoechea are grateful for financial support from Research Group B at the 10.13039/501100003451University of the Basque Country (Ref. IT1777-22).

## CRediT authorship contribution statement

**I. Galarraga:** Writing – review & editing, Writing – original draft, Project administration, Methodology, Investigation, Funding acquisition, Formal analysis, Data curation, Conceptualization. **L.M. Abadie:** Writing – review & editing, Writing – original draft, Methodology, Investigation, Formal analysis, Data curation, Conceptualization. **T. Standfuss:** Writing – review & editing, Writing – original draft, Methodology, Funding acquisition, Formal analysis, Data curation, Conceptualization. **I. Ruiz-Gauna:** Writing – review & editing, Writing – original draft, Methodology, Investigation, Funding acquisition, Formal analysis, Data curation, Conceptualization. **N. Goicoechea:** Writing – review & editing, Validation, Supervision, Methodology, Formal analysis.

## Declaration of competing interest

The authors declare the following financial interests/personal relationships which may be considered as potential competing interests: Ibon Galarraga reports financial support was provided by FABEC. Thomas Standfuss reports financial support was provided by FABEC. If there are other authors, they declare that they have no known competing financial interests or personal relationships that could have appeared to influence the work reported in this paper.

## Figures and Tables

**Table A1:** 

DSNA	2015	2016	2017	2018	2019
**A**	0.0004539	2.21E-11	5.24E-41	1.41E-42	2.59E-23
**B**	1.798	3.667	11.04	11.47	6.63
**Goodness- of-fit**	SSE: 1.128e+10 R-square: 0.05479 Adj. R-square: 0.05219 RMSE: 5574	SSE: 1.271e+10 R-square: 0.205 Adj. R-square: 0.2028 RMSE: 5910	SSE: 7.369e+09 R-square: 0.5749 Adj. R-square: 0.5738 RMSE: 4506	SSE: 2.787e+10 R-square: 0.5546 Adj. R-square: 0.5534 RMSE: 8763	SSE: 2.937e+10 R-square: 0.2731 Adj. R-square: 0.2711 RMSE: 8995

**Table A2:** 

ANSCR	2015	2016	2017	2018	2019
**A**	5.22E-30	9.56E-07	4.60E-05	6.21E-14	1.01E-11
**B**	9.07	2.043	1.813	4.764	4.025
**Goodness- of-fit**	SSE: 4.274e+06 R-square: 0.01266 Adj. R-square: 0.009935 RMSE: 108.5	SSE: 1.206e+06 R-square: 0.002108 Adj. R-square: 0.0006332 RMSE: 57.57	SSE: 1.233e+07 R-square: 0.009528 Adj. R-square: 0.0068 RMSE: 184.3	SSE: 4.166e+08 R-square: 0.2781 Adj. R-square: 0.2761 RMSE: 1071	SSE: 1.684e+08 R-square: 0.1107 Adj. R-square: 0.1083 RMSE: 681.1

**Table A3:** 

Austro Control	2015	2016	2017	2018	2019
**A**	8.00E-22	6.81E-32	1.15E-27	1.80E-22	1.92E-13
**B**	6.664	9.451	8.397	7.064	4.618
**Goodness- of-fit**	SSE: 4.844e+07 R-square: 0.03635 Adj. R-square: 0.03369 RMSE: 365.3	SSE: 7.71e+06 R-square: 0.04768 Adj. R-square: 0.3655 RMSE: 0.04507	SSE: 1.438e+08 R-square: 0.1382 Adj. R-square: 0.1358 RMSE: 629.5	SSE: 1.533e+09 R-square: 0.224 Adj. R-square: 0.2218 RMSE: 2055	SSE: 5.548e+09 R-square: 0.2568 Adj. R-square: 0.2548 RMSE: 3910

**Table A4:** 

Croatia Control	2015	2016	2017	2018	2019
**A**	8.43E-10	2.75E-24	6.08E-09	3.66E-13	4.16E-12
**B**	3.691	7.622	3.144	4.598	4.307
**Goodness- of-fit**	SSE: 4.74e+08 R-square: 0.2909 Adj. R-square: 0.289 RMSE: 1143	SSE: 3.408e+06 R-square: 0.196 Adj. R-square: 0.1938 RMSE: 96.76	SSE: 3.65e+07 R-square: 0.1022 Adj. R-square: 0.0997 RMSE: 317.1	SSE: 2.585e+08 R-square: 0.4947 Adj. R-square: 0.4933 RMSE: 843.8	SSE: 7.365e+08 R-square: 0.4864 Adj. R-square: 0.485 RMSE: 1424

**Table A5:** 

DCAC	2015	2016	2017	2018	2019
**A**	9.76E-06	8.09E-23	1.05E-16	1.78E-21	8.10E-16
**B**	2.825	8.279	6.281	7.757	5.914
**Goodness- of-fit**	SSE: 1.614e+09 R-square: 0.2346 Adj. R-square: 0.2325 RMSE: 2109	SSE: 2.515e+08 R-square: 0.3284 Adj. R-square: 0.3265 RMSE: 831.1	SSE: 9.876e+08 R-square: 0.3821 Adj. R-square: 0.3804 RMSE: 1649	SSE: 1.307e+09 R-square: 0.4078 Adj. R-square: 0.4061 RMSE: 1897	SSE: 1.082e+09 R-square: 0.3157 Adj. R-square: 0.3138 RMSE: 1727

**Table A6:** 

MUAC	2015	2016	2017	2018	2019
**A**	6.07E-23	2.80E-17	1.22E-21	3.58E-28	3.42E-22
**B**	6.858	5.328	6.528	8.283	6.494
**Goodness- of-fit**	SSE: 5.482e+08 R-square: 0.27 Adj. R-square: 0.268 RMSE: 1229	SSE: 8.638e+08 R-square: 0.2008 Adj. R-square: 0.1987 RMSE: 1541	SSE: 3.216e+09 R-square: 0.193 Adj. R-square: 0.1908 RMSE: 2976	SSE: 2.972e+09 R-square: 0.252 Adj. R-square: 0.25 RMSE: 2861	SSE: 1.734e+08 R-square: 0.1257 Adj. R-square: 0.1233 RMSE: 691.1

**Table A7:** 

NATS	2015	2016	2017	2018	2019
**A**	2.20E-20	1.23E-20	1.72E-20	2.13E-16	2.27E-37
**B**	5.795	6.003	5.873	4.89	10.24
**Goodness- of-fit**	SSE: 2.644e+08 R-square: 0.04788 Adj. R-square: 0.04526 RMSE: 853.5	SSE: 1.764e+09 R-square: 0.1274 Adj. R-square: 0.125 RMSE: 2201	SSE: 4.358e+08 R-square: 0.1678 Adj. R-square: 0.1655 RMSE: 1096	SSE: 1.919e+09 R-square: 0.1286 Adj. R-square: 0.1262 RMSE: 2299	SSE: 1.116e+09 R-square: 0.2265 Adj. R-square: 0.2244 RMSE: 1753

**Table A8:** 

NAV Portugal	2015	2016	2017	2018	2019
**A**	8.97E-16	1.24E-20	5184	1.88E-44	2.77E-53
**B**	5.671	6.994	−0.383	14.13	16.79
**Goodness- of-fit**	SSE: 3.714e+08 R-square: 0.1661 Adj. R-square: 0.1638 RMSE: 1011	SSE: 2.386e+08 R-square: 0.05541 Adj. R-square: 0.05281 RMSE: 809.6	SSE: 9.036e+08 R-square: 0.0001024 Adj. R-square: 0.0026 RMSE: 1578	SSE: 3.297e+08 R-square: 0.03581 Adj. R-square: 0.03315 RMSE: 953	SSE: 3.448e+08 R-square: 0.08062 Adj. R-square: 0.07808 RMSE: 974.6

**Table A9:** 

skeyes	2015	2016	2017	2018	2019
**A**	9.21 E+04	2.53E-12	1.58E-23	8.05E-20	6.64E-08
**B**	−0.8213	4.479	7.658	6.55	3.183
**Goodness- of-fit**	SSE: 7.835e+08 R-square: 0.0002938 Adj. R-square: 0.00246 RMSE: 1469	SSE: 7.634e+08 R-square: 0.09191 Adj. R-square: 0.08941 RMSE: 1448	SSE: 8.686e+07 R-square: 0.07014 Adj. R-square: 0.0676 RMSE: 489.2	SSE: 1.681e+08 R-square: 0.05742 Adj. R-square: 0.05482 RMSE: 680.5	SSE: 1.476e+09 R-square: 0.1007 Adj. R-square: 0.0982 RMSE: 2016

**Table A10:** 

skyguide	2015	2016	2017	2018	2019
**A**	1.59E-09	5.03E-20	5.51E-14	2.66E-40	6.06E-33
**B**	3.16	6.126	4.49	11.79	9.724
**Goodness- of-fit**	SSE: 4.829e+07 R-square: 0.05903 Adj. R-square: 0.05644 RMSE: 364.7	SSE: 1.302e+08 R-square: 0.07599 Adj. R-square: 0.07345 RMSE: 598.1	SSE: 1.583e+08 R-square: 0.1522 Adj. R-square: 0.1499 RMSE: 660.3	SSE: 4.993e+08 R-square: 0.2246 Adj. R-square: 0.2224 RMSE: 1173	SSE: 5.034e+08 R-square: 0.1424 Adj. R-square: 0.1401 RMSE: 1178

**Fig. A1:**
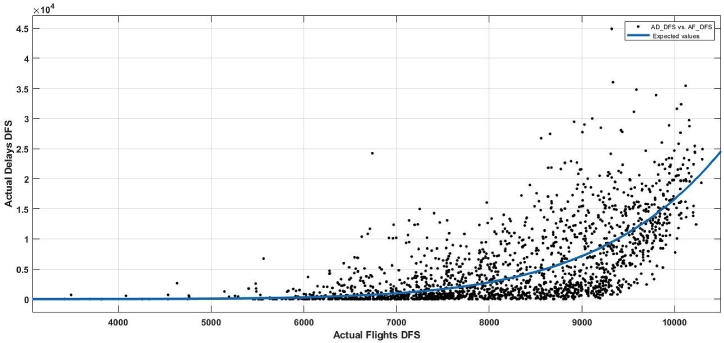
Delays and flights data for DFS (2015–2019)

**Table B1:** ATFM delay ranges and weighted costs (average and per minute) in 2010 Euros

Delay range (min)	01–04	05–14	15–29	30–59	60–89	90–119	120–179	180–239	240–299	300+
Average total cost (Euros)	32	210	870	3870	11,940	25,560	39,710	53,220	70,450	80,270
Average cost per minute (Euros)	12,8	22,11	39,55	86,97	160,27	244,59	265,62	254,03	261,41	267,57

Source: own work based on Table J5 in Cook & Tanner (2011).

**Table C1:** Actual and Backcasted Flights for each year (2015–2019) and ANSP

	2015		2016		2017		2018		2019	
	Actual Flights	BackcastedFlights	Actual Flights	Backcasted Flights	Actual Flights	BackcastedFlights	Actual Flights	BackcastedFlights	Actual Flights	BackcastedFlights
DFS	2,818,110	2,769,872	2,892,868	2,814,708	2,994,472	2,824,775	3,113,468	2,844,906	3,118,176	2,867,783
DSNA	2,918,465	2,871,869	3,051,154	2,922,527	3,173,063	2,924,473	3,257,894	2,942,983	3,302,045	2,964,414
ANSCR	730,979	723,356	781,615	750,690	797,657	771,190	856,742	792,666	850,179	817,070
AustroControl	915,007	893,176	918,769	920,289	994,781	943,528	1,063,825	966,768	1,108,735	994,655
CroatiaControl	530,607	531,908	533,791	549,972	581,327	567,034	640,384	582,088	707,995	602,160
DCAC	319,091	311,335	322,214	324,350	359,540	331,357	393,558	342,369	411,460	352,380
MUAC	1,702,263	1,681,924	1,779,969	1,711,533	1,848,581	1,720,417	1,872,690	1,736,235	1,862,754	1,752,114
NATS	2,268,666	2,236,383	2,399,723	2,280,253	2,490,666	2,317,298	2,514,044	2,359,217	2,536,427	2,403,087
NAV Portugal	501,873	491,479	556,204	502,424	610,028	511,377	630,321	522,321	647,617	533,265
Skeyes	591,480	570,567	595,248	580,010	628,705	581,999	649,574	587,466	639,865	592,933
Skyguide	1,184,665	1,167,171	1,205,751	1,182,959	1,242,610	1,186,342	1,303,816	1,196,491	1,310,481	1,207,768

**Table D1:** Difference between Actual and Backcasted CRSTMP Delay Minutes for all years (2015–2019) and ANSP by applying clustered analysis and non-clustered daily data

Actual CRSTMP Delay Minutes	2015			2016			2017			2018			2019		
	Actual	Backcasted		Actual	Backcasted		Actual	Backcasted		Actual	Backcasted		Actual	Backcasted	
		Clust.	Non-clust		Clust.	Non-clust		Clust.	Non-clust		Clust.	Non-clust		Clust.	Non-clust
DFS	319,775	296,978	301,959	661,392	531,382	521,934	1,472,063	1,044,781	989,609	3,845,332	2,603,777	2,460,230	3,702,239	2,816,076	2,740,061
DSNA	1,773,854	1,675,028	1,711,398	2,318,769	1,893,577	1,899,507	1,945,697	1,023,197	729,327	3,513,040	1,616,542	1,008,839	2,513,994	1,274,618	1,211,457
ANSCR	3654	3472	3553	2215	2997	2106	19,126	18,746	18,287	326,359	237,220	226,777	149,803	122,719	128,085
AustroControl	21,679	20,312	20,137	8481		9580	70,556	46,391	47,785	361,210	146,437	191,572	1,029,493	557,350	655,005
CroatiaControl	203,834		229,445	8505		11,155	33,541	31,098	34,196	219,402	113,337	153,703	391,689	126,627	212,131
DCAC	779,141	708,944	735,989	203,423		177,262	397,175	232,862	244,996	428,116	138,747	150,610	466,441	187,284	188,799
MUAC	394,377	370,682	371,188	517,891	434,450	421,988	824,785	590,675	524,365	927,974	560,024	492,834	176,905	112,787	118,279
NATS	94,582	85,753	89,818	494,652	380,901	350,795	261,604	168,596	171,990	532,029	181,214	394,107	362,126	233,902	200,452
NAV Portugal	239,497	213,550	219,168	105,010	44,996	51,646	110,699	101,676	118,435	110,307	38,329	5662	150,330	71,025	3886
Skeyes	80,122	68,351	81,971	261,878	236,153	237,739	56,614	31,809	31,068	79,834	36,024	41,298	542,010	424,291	430,872
Skyguide	78,068	72,899	74,101	92,402	84,849	79,201	173,472	136,786	140,274	255,952	110,949	80,947	191,416	97,519	81,293

**Table E1:** Actual and backcasted CRSTMP delay costs using an average value of €104 per minute and costs due to underestimation

Backcasted CRSTMP Delay Costs	2015		2016		2017		2018		2019	
	Actual	Backcasted	Actual	Backcasted	Actual	Backcasted	Actual	Backcasted	Actual	Backcasted
DFS	31,977,500	29,697,815	66,139,200	53,138,193	150,150,426	106,567,638	399,914,528	270,792,783	392,437,334	298,504,081
DSNA	177,385,400	167,502,765	231,876,900	189,357,655	198,461,094	104,366,065	365,356,160	168,120,392	266,483,364	135,109,513
ANSCR	365,400	347,239	221,500	299,703	1,950,852	1,912,090	33,941,336	24,670,913	15,879,118	13,008,255
Austro Control	2,167,900	2,031,173	848,100		7,196,712	4,731,920	37,565,840	15,229,430	109,126,258	59,079,049
Croatia Control	20,383,400		850,500		3,421,182	3,172,019	22,817,808	11,787,056	41,519,034	13,422,420
DCAC	77,914,100	70,894,351	20,342,300		40,511,850	23,751,874	44,524,064	14,429,717	49,442,746	19,852,084
MUAC	39,437,700	37,068,157	51,789,100	43,445,036	84,128,070	60,248,827	96,509,296	58,242,542	18,751,930	11,955,450
NATS	9,458,200	8,575,335	49,465,200	38,090,076	26,683,608	17,196,815	55,331,016	18,846,238	38,385,356	24,793,654
NAV Port	23,949,700	21,354,986	10,501,000	4,499,606	11,291,298	10,370,996	11,471,928	3,986,257	15,934,980	7,528,638
Skeyes	8,012,200	6,835,067	26,187,800	23,615,279	5,774,628	3,244,568	8,302,736	3,746,520	57,453,060	44,974,864
Skyguide	7,806,800	7,289,863	9,240,200	8,484,886	17,694,144	13,952,186	26,619,008	11,538,731	20,290,096	10,337,041

**Table E2:** Actual and backcasted CRSTMP delay costs using the cost functions and costs due to underestimation

Backcasted CRSTMP Delay Costs	2015		2016		2017		2018		2019	
	Actual	Backcasted	Actual	Backcasted	Actual	Backcasted	Actual	Backcasted	Actual	Backcasted
DFS	13,980,893	8,129,013	22,549,084	15,400,684	44,355,979	27,037,372	130,774,309	78,469,862	113,281,691	78,994,108
DSNA	74,380,101	58,637,135	95,469,422	68,592,464	77,366,063	26,695,568	163,119,784	42,001,694	108,660,738	41,644,966
ANSCR	145,805	3667	74,356	1005	591,257	87,198	10,818,768	5,630,954	4,873,742	2,401,691
Austro Control	855,132	101,586	334,732	30,908	2,696,482	705,004	16,441,992	5,652,566	42,465,450	18,849,489
Croatia Control	7,552,584	4,926,504	258,778	92,047	1,116,602	307,025	8,048,471	3,954,264	13,422,042	5,272,184
DCAC	40,908,904	30,343,867	8,561,272	5,759,463	19,963,876	8,985,264	24,284,509	6,192,055	23,689,638	6,908,410
MUAC	12,445,084	9,221,790	15,042,782	10,127,939	26,060,802	13,756,832	34,378,003	14,964,292	5,057,959	2,377,923
NATS	4,757,683	1,284,439	23,236,117	10,448,234	10,767,439	4,664,614	23,050,358	12,367,854	17,299,777	6,339,619
NAV Port	9,037,993	4,672,693	4,516,956	639,011	7,775,264	963,665	4,849,732	88,904	6,911,808	88,289
Skeyes	6,063,739	256,894	12,386,460	5,200,075	2,192,130	393,824	3,227,859	483,669	22,629,127	11,703,863
Skyguide	3,205,874	1,823,385	4,050,268	2,542,300	6,247,446	4,123,260	10,474,932	3,025,248	7,830,588	2,839,243

## Appendix B AFTM delay costs

These are 2010 values, but we need 2015 values. Considering that the network average cost of ATFM delay was 100 Euros/minute in 2017 values, as explained above, and 81 Euros/minute in 2010 values, there was an increase of 23.45%. By applying this percentage increase to the figures in the last row of Table B1, an approximation of ATFM delay cost per minute in 2015 values can be calculated.

## Footnotes

11 Example: If the forecast is 5% below the actual value, we deduct 5% of the flights for each day in order to maintain the annual distribution of flights.

## Footnotes

1 https://www.eurocontrol.int/.

2 This is the Eurocontrol Statistics and Forecast Service. It provides statistics and forecasts on air traffic in Europe and to monitor and analyse trends in the air transport industry.

3 The growth forecast was 3.6% under the high-growth scenario and 1.5% under the low-growth scenario.

4 Other studies have recently examined and applied different quantitative methods and simulation approaches for forecasting traffic demand, but none of them applies backcasting for determining the cost of delays ([[19], [20], [21], [22]] Tascón & Díaz Olariaga, 2021).

5 Reporting Period 2 of the Single European Sky (SES) Performance Scheme for the EU and associated states.

6 STATFOR also provides an autumn report, but it is available for fewer years. For the sake of consistency, we only use data from spring reports.

7 They refer to Air Traffic Control (ATC) Capacity (delays due to traffic demand exceeding declared or expected ATC capacity), ATC Routings (delays due to network solutions/scenarios used to balance traffic demand and capacity), ATC Staffing (delays due to unplanned staff shortages that reduce expected capacity), ATC Equipment (delays due to the non-availability or degradation of equipment used to provide an ATC service that reduce expected or declared capacity), Airspace Management (delays following changes in airspace/route availability due to small-scale military activity that reduce expected or declared capacity) and Special Events (delays due to major sporting, government or social events that reduce planned, declared or expected capacity).

8 [30] also focuses on understanding these costs, albeit from a different perspective. A stochastic modelling method is proposed to estimate future air traffic, delays and the cost of future delays. The model provides a better understanding of how the probabilities of air traffic and delays are distributed, and thus of what the full distribution of delay costs may look like.

9 We do not refer to the ATFM regulations by NM.

10 We use daily data, so probabilities cannot be calculated using raw data.

12 For illustrative purposes we have plotted the delay data for DFS in Figure A1 in Appendix A.

13 Note that coefficients a and b were determined using scale and offset parameters.
